# DNA/BSA Binding Affinity and Cytotoxicity of Dinuclear Palladium(II) Complexes with Amino Acids as Ligands

**DOI:** 10.3390/molecules30071534

**Published:** 2025-03-30

**Authors:** Stefan Jakovljevic, Petar Canovic, Marko Spasic, Marija Zivkovic, Milan Zaric, Radica Zivkovic Zaric, Andjela Franich, Snezana Rajkovic, Zeljko Todorovic, Nenad Relic, Milos Zivic, Nikola Mirkovic

**Affiliations:** 1 Department of Surgery, Faculty of Medical Sciences, University of Kragujevac, Svetozar Markovic Street 69, 34000 Kragujevac, Serbia; stefan_jakov87@yahoo.com (S.J.); drnikolamirkovic@gmail.com (N.M.); 2 Department of General Surgery, University Clinical Center Kragujevac, Zmaj Jovina Street 30, 34000 Kragujevac, Serbia; 3 Department of Biochemistry, Faculty of Medical Sciences, University of Kragujevac, Svetozar Markovic Street 69, 34000 Kragujevac, Serbia; petar.c89@gmail.com (P.C.); zaricmilan@gmail.com (M.Z.); 4 Department of Pharmacy, Faculty of Medical Sciences, University of Kragujevac, Svetozar Markovic Street 69, 34000 Kragujevac, Serbia; 5 Department of Pharmacology and Toxicology, Faculty of Medical Sciences, University of Kragujevac, Svetozar Markovic Street 69, 34000 Kragujevac, Serbia; radica_zivkovic@yahoo.com; 6 Department of Clinical Pharmacology, University Clinical Center Kragujevac, Zmaj Jovina Street 30, 34000 Kragujevac, Serbia; 7 Department of Chemistry, Faculty of Sciences, University of Kragujevac, Radoje Domanovic Street 12, 34000 Kragujevac, Serbia; andjela.franich@pmf.kg.ac.rs (A.F.); snezana.rajkovic@pmf.kg.ac.rs (S.R.); 8 Department of Internal Medicine, Faculty of Medical Sciences, University of Kragujevac, Svetozar Markovic Street 69, 34000 Kragujevac, Serbia; todorovic_zeljko@hotmail.com; 9 Clinic for Hematology, University Clinical Center Kragujevac, Zmaj Jovina Street 30, 34000 Kragujevac, Serbia; 10 Department of Otorhinolaryngology, Faculty of Medical Sciences, University of Kragujevac, Svetozar Markovic Street 69, 34000 Kragujevac, Serbia; nenadrelicmd@gmail.com; 11 Clinic of Otorhinolaryngology, University Clinical Center Kragujevac, Zmaj Jovina Street 30, 34000 Kragujevac, Serbia; 12 Department of Dentistry, Faculty of Medical Sciences, University of Kragujevac, Svetozar Markovic Street 69, 34000 Kragujevac, Serbia; zivicmilos5@gmail.com; 13 Department of Maxillofacial Surgery, Clinic of Otorhinolaryngology, University Clinical Center Kragujevac, 34000 Kragujevac, Serbia; 14 Department of Vascular Surgery Center, University Clinical Center Kragujevac, Zmaj Jovina Street 30, 34000 Kragujevac, Serbia

**Keywords:** palladium, pyrazine, amino acids, DNA, albumin, cytotoxicity, apoptosis

## Abstract

This study investigates the synthesis, characterization, and cytotoxicity of dinuclear palladium(II) complexes with glycine (Pd1), alanine (Pd2), and methionine (Pd3) as ligands. UV-Vis and fluorescence spectroscopy were used to investigate the complexes’ interactions with calf thymus DNA (CT-DNA) and bovine serum albumin. The obtained measurements demonstrate that Pd1 and Pd2 have stronger binding affinities for CT-DNA compared to Pd3, with Pd3 exhibiting the most significant cytotoxicity against the MDA-MB-231 cancer cell line. The binding behavior was quantified by calculating intrinsic binding constants (K_b_) and Stern–Volmer constants (K_sv_), showing that Pd1 and Pd2 interact more effectively with DNA, possibly due to less steric hindrance in their chelation. Cytotoxic activity was evaluated using an MTT assay, and the results confirm that Pd3, with methionine as the ligand, exhibited superior antitumor effects, inducing apoptosis through caspase-3 activation. The complexes also showed a strong affinity for BSA, indicating their potential for biological interaction. These discoveries shed light on the processes of palladium(II) complexes in biological systems, highlighting their DNA and protein-binding capabilities, as well as their anticancer potential. Further research is required to explore their pharmacokinetics and possible clinical applications.

## 1. Introduction

Coordination chemistry enables the development of new medicinal and diagnostic drugs by controlling the solubility, reactivity, steric effects, and shape of the metal complex by simply changing or modifying the ligand surrounding the main metal ion. Small changes in molecular structure can result in significant alterations in biological function. This results in numerous classes of putative anticancer platinum(II) and palladium(II) complexes [1,2]. A number of palladium(II) complexes with ligands derived from pyridine, quinoline, pyrazole, and 1,10-phenanthroline have demonstrated highly promising antitumor properties [3,4,5]. The stability of the Pd(II)–ligand bond must be taken into account when synthesizing potentially active antitumor palladium(II) complexes because, under physiological conditions, palladium(II) complexes hydrolyze very quickly so, in vivo, they react very quickly with a large number of biomolecules, especially with proteins, which prevents their transport to DNA [6,7,8]. As it is proven that dinuclear palladium(II) complexes have in vitro and in vivo antitumor properties similar to cisplatin, recent research is based on synthetic complexes that contain two or more palladium(II) ions [9,10,11,12].

Antitumor activity in cisplatin treatments is reflected in the formation of covalent bonds with the N-7 atoms of guanine groups, resulting in the bending of DNA molecules and unwinding of the helix, thereby preventing replication and transcription of the DNA molecules, followed by cell apoptosis [13]. As a result, platinum(II) and palladium(II) analogs have attracted interest in the treatment of a variety of malignancies, and their method of interaction with recognized biomolecules, such as DNA and serum albumin, has been thoroughly investigated [14]. Recently, we examined the binding affinities of dinuclear palladium(II) complexes comprising various benzodiazine-bridging ligands and pyridine-based bridging ligands to CT-DNA and BSA biomolecules [5,15]. It was shown that the position of the nitrogen-donor atoms in the bridging ligands plays a crucial effect in the binding of dinuclear palladium(II) complexes to biomolecules, as well as their cytotoxic action against the examined tumor cell lines. Furthermore, several of the studied complexes were more cytotoxic against A549 cells than cisplatin, although all complexes had a greater apoptotic effect on murine lung cancer LLC1 cells than cisplatin.

Cancer is a significant global health concern and the second leading cause of death from non-infectious diseases. Studies indicate that over 40% of adults will be diagnosed with some form of cancer during their lifetime [16]. Apoptosis, a programmed cell death mechanism, is crucial for controlling cell proliferation. Defects in apoptosis can lead to carcinogenesis, playing a key role in tumor growth and resistance to therapies [17]. Changes in the expression of critical apoptosis regulatory proteins, including antiapoptotic bcl-2 and proapoptotic BAX, are required to activate apoptosis [17,18]. These alterations can activate caspase-3, a key component in the apoptotic process [19]. Understanding apoptotic pathways provides prospects for the development of novel therapeutics. Cisplatin, a commonly used anticancer drug, has limits due to serious toxic side effects, such as nephrotoxicity, neurotoxicity, ototoxicity, and resistance after long-term treatment [20]. Consequently, new metal-containing complexes, including platinum, palladium, ruthenium, and gold, are being explored as potential antitumor agents.

In order to investigate how the nature of bidentate-coordinated ligands in dinuclear palladium(II) complexes influence the binding and cytotoxic properties of the complexes in the present study, we synthesized three dinuclear complexes [{Pd(Gly*-N,O*)Cl}_2_(*µ*-pz)] (Pd1), [{Pd(Ala*-N,O*)Cl}_2_(*µ*-pz)] (Pd2), and [{Pd(Met-*S,N*)Cl}_2_(*µ*-pz)](NO_3_)_2_ (Pd3), where Gly is glycine, Ala is alanine, Met is methionine, and pz is pyrazine. The complexes were structurally characterized by elemental analysis, ^1^H NMR, UV-Vis, and IR spectroscopy. The palladium(II) complexes’ reactions with DNA can reveal the mechanism of their anticancer activity, as well as their ability to react as drugs. In agreement with this, we studied the interactions of Pd1–Pd3 complexes with CT-DNA using UV-Vis and fluorescence spectroscopy. Bovin serum albumin (BAS), an important biomolecule containing the amino acids tryptophan, tyrosine, and phenylalanine, can interact with palladium(II) complexes. The complexes’ manner of interaction and biological preferences can be predicted based on their binding possibilities to BSA. The interaction of Pd1–Pd3 complexes with BSA was studied using fluorescence spectroscopy. Finally, the cytotoxic effect of the newly synthesized palladium complexes on two human breast cancer cell lines was examined using an MTT assay, while the mechanism of the cytotoxic effect was examined using flow cytometry.

## 2. Results and Discussion

### 2.1. Synthesis and Characterization of [{Pd(Gly-N,O)Cl}_2_(µ-pz)], [{Pd(Ala-N,O)Cl}_2_(µ-pz)], and [{Pd(Met-S,N)Cl}_2_(µ-pz)](NO_3_)_2_ Complexes

**Figure 1:**
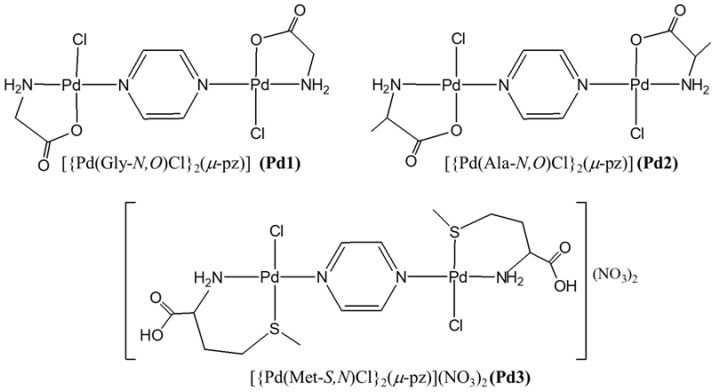
The structural formulas of Pd1–Pd3 complexes.

Three dinuclear complexes [{Pd(Gly*-N,O*)Cl}_2_(*µ*-pz)] (Pd1), [{Pd(Ala*-N,O*)Cl}_2_(*µ*-pz)] (Pd2), and [{Pd(Met-*S,N*)Cl}_2_(*µ*-pz)](NO_3_)_2_ (Pd3) (Figure 1), where glycine (Gly), alanine (Ala), and methionine (Met) are bidentate-coordinated amino acids, while pyrazine (pz) is the bridging ligand, were synthesized and characterized using the results of elemental microanalysis, ^1^H NMR, UV-Vis, and IR spectroscopy. The reaction of K[Pd(Gly-*N,O*)Cl_2_], K[Pd(Ala-*N,O*)Cl_2_]·2H_2_O, and [Pd(Met-*S,N*)Cl_2_] with an equimolar amount of AgNO_3_ in a dmf solution was used to obtain the monoactivated [Pd(Gly-*N,O*)Cl(dmf)], [Pd(Ala-*N,O*)Cl(dmf)], and [Pd(Met-*S,N*)Cl(dmf)]^+^ species. After removal of the precipitated AgCl, the dmf solution of pz ligand was added into the solution of [Pd(Gly-*N,O*)Cl(dmf)], [Pd(Ala-*N,O*)Cl(dmf)], and [Pd(Met-*S,N*)Cl(dmf)]^+^.

To obtain the Pd1–Pd3 complexes, the solution was stirring at room temperature and reduced under vacuum to a small volume. Dichloromethane was then added. Our attempts to crystallize these compounds from their amorphous powders with various solvents failed.

^1^H NMR spectroscopy: The ^1^H NMR spectra of pyrazine, glycine, L-alanine, and L-methionine, as well as the spectra of Pd1–Pd3 complexes, were recorded in D_2_O as a solvent (Figure S1). After the bidentate coordination of the amino acids to the palladium(II) ion, the signal of the associated protons moves to a lower field, resulting in a greater chemical shift (Table 1).

Also, after coordination of the bridging pyrazine ligand to palladium(II) ion, the singlet originating from the four equivalent protons of the pyrazine ring shifts to the lower field by Δδ = 0.17 ppm for Pd1, Δδ = 0.26 ppm for Pd2, and Δδ = 0.23 ppm for Pd3. These results are in agreement with the data obtained on the basis of ^1^H NMR spectroscopy for the [{Pt(en)Cl}_2_(*μ*-pz)]^2+^ [21] and [{Pd(en)Cl}_2_(*μ*-pz)]^2+^ [22] complexes.

UV–Vis spectroscopy: The UV–Vis spectra of Pd1–Pd3 display intense bands at 260 nm for Pd1 and Pd2 and 259 for Pd3 complex (Figure S2) and have a bathochromic shift after pyrazine coordination due to π^….^π electronic transitions in the pyrazine ligand. The Materials and Methods section contains the maximum absorption wavelengths (λ_max_, nm) and molar extinction coefficients (ε, M^−1^cm^−1^) for these complexes.

IR spectroscopy: The IR spectra of the dinuclear Pd1–Pd3 (Figure S3) complexes measured in the 4000–450 cm^−1^ range contain bands attributable to bridging ligands as well as bidentate-coordinated amino acids. The bands between 3310 and 3054 cm^−1^ correspond to the asymmetric and symmetric stretching frequencies of the bidentate-coordinated NH_2_ group of Gly, Ala, and Met. The bands in the range of 1632–1573 cm^−1^ correspond to the ring stretching frequencies of ν(C=C) and ν(C=N) in the pz ligand. The ν(COO^−^) band at 1686 cm^−1^ for Pd1 and 1687 cm^−1^ for Pd2 confirms the bidentate coordination of glycine (Pd1) and alanine (Pd2) via NH_2_ and a deprotonated carboxyl group on palladium(II). The IR spectra of the Pd3 complex show a ν(COOH) band at 1723 cm^−1^, indicating that the carboxylate group in this complex is free and protonated. The very prominent band at 1378–1308 cm^−1^ in the IR spectra of the Pd3 complex reveals the presence of NO_3_^−^ ions in the exterior coordination sphere [22].

### 2.2. Analysis of DNA Binding and Interactions

**Figure 2:**
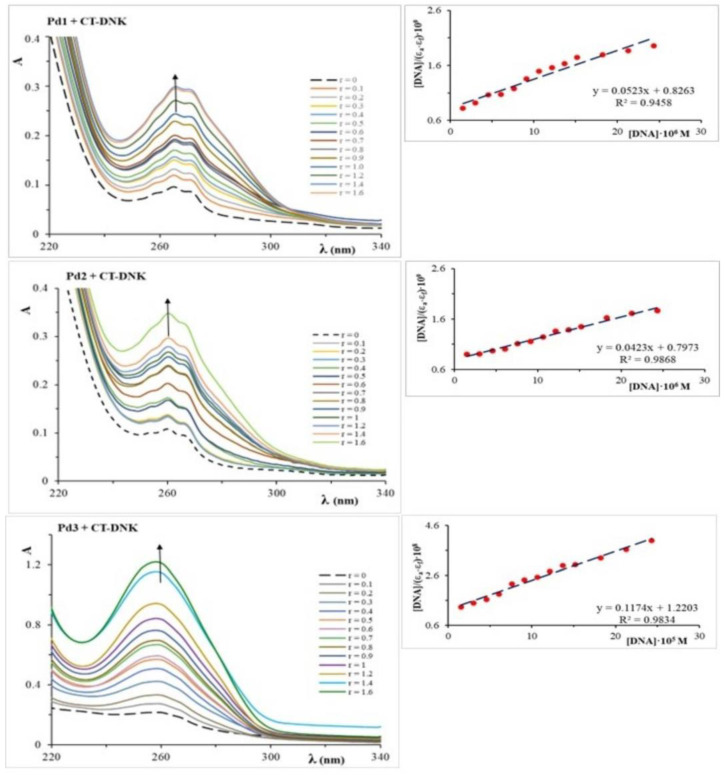
UV−Vis spectra of Pd1–Pd3 complexes in the absence and presence of increasing amounts of CT–DNA in 10 mM PBS at pH 7.40 and 310 K.

UV-Vis spectroscopy is a method for determining the mechanism of coordination or interaction of metal complexes with deoxyribonucleic acid (DNA) molecules. Transition metal complexes have both covalent and non-covalent interactions with DNA [23,24]. Covalent bonds are formed when DNA molecules coordinate, typically via the N7 atom from guanine to a metal ion, whereas non-covalent interactions include intercalation, hydrogen bond formation, and electrostatic interactions. After the metal complex interacts with DNA, the absorbance intensity can either drop (hypochromic impact) or rise (hyperchromic effect). A bathochromic or hypochromic shift can also occur at the absorption maximum. UV-Vis spectroscopy results can be used to calculate the intrinsic binding (stability) constants of DNA to metal ions. This study examined the UV-Vis spectra of Pd1–Pd3 complexes in the absence and presence of varying CT-DNA concentrations ([Pd(II)]/[CT-DNA] = 0.0–1.6) (Figure 2). The addition of CT-DNA to the solution of dinuclear palladium(II) complexes results in a hyperchromic effect, indicating that the complexes interact with CT-DNA. The hyperchromic effect implies that the Pd1 and Pd2 complexes with CT-DNA achieve π→π* interactions as a result of the interaction of the aromatic rings of the pyrazine ligand in palladium(II) complexes and the base in CT-DNA. As the Pd3 complex has a +2 charge, it can also interact electrostatically, in addition to π→π* [25]. The binding constants (K_b_) were determined using the equation provided in the Materials and Methods section of this paper (Section 3.3.1) and the change in absorbance at the corresponding wavelength following the addition of CT-DNA (Figure 2). 

The obtained data for K_b_ are shown in Table 2. According to K_b_, we conclude that interactions exist between the tested palladium(II) complexes and CT-DNA, which are inherent in intercalators. The Kb values of the Pd1–Pd3 complexes are lower than the binding constant K_b_ of the standard intercalator ethidium–bromide to DNA, which is 1.23·10^5^ M^−1^. This suggests that the interactions of the examined complexes are weaker compared to EtBr [26]. The K_b_ values for the Pd1 and Pd2 complexes are approximately six times higher than those for Pd3, indicating that the Pd1 and Pd2 complexes interact with CT-DNA with a higher affinity than the Pd3 complex.

As the bidentate-coordinated five-membered glycine and alanine rings have a smaller steric influence than the six-membered methionine ring, the Pd1 and Pd2 complexes have larger numerical values of K_b_ than the Pd3 complex. The Pd3 complex’s six-membered chelate ring changes conformation, which makes its interactions with DNA weaker. Furthermore, this complex is in a cationic form (+2), which enables it to bind electrostatically with the negatively charged DNA backbone in contrast to the neutral Pd1 and Pd2 complexes [27]. The negative Gibbs energy values indicate the spontaneity of the interaction of dinuclear palladium(II) complexes with CT-DNA (Table 2).

**Figure 3:**
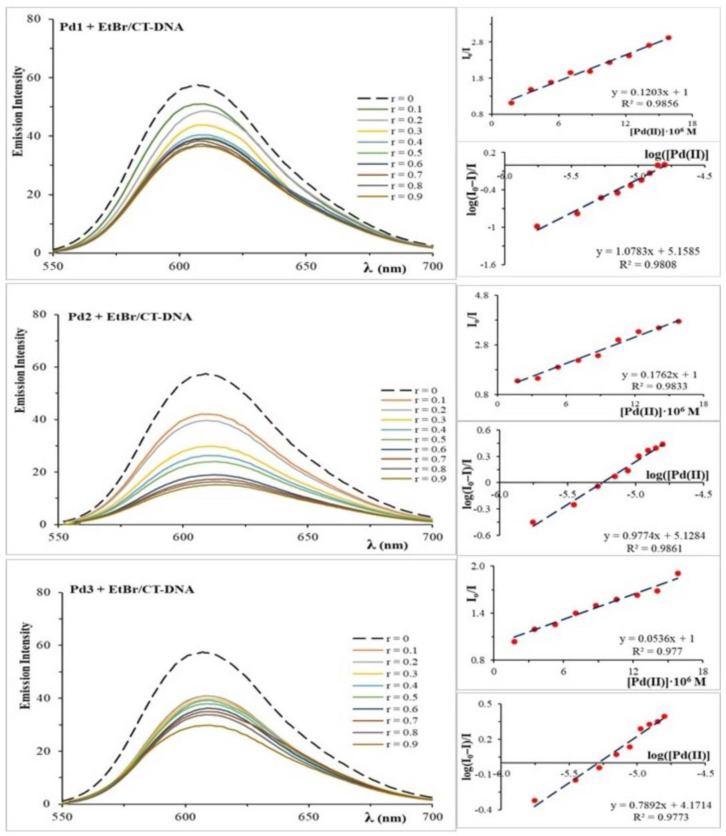
Emission spectra of the EtBr/CT–DNA system in the absence and presence of increasing amounts of Pd1–Pd3 complexes in 10 mM PBS at pH 7.40 and 298 K.

The interactions between the dinuclear palladium(II) complexes and CT-DNA molecules were studied using emission fluorescence spectroscopy. Ethidium bromide (EtBr) was employed as the standard intercalator in these assays. Changes in the EtBr/CT-DNA emission spectra following the addition of the complex solution were used to investigate the complex’s interactions with DNA. After adding the complex, the emission spectra may show a decrease or increase in fluorescence emission, indicating that the complex replaces EtBr, forming a new adduct CT-DNA/Pd(II) complex [28]. Figure 3 displays the emission spectra of EtBr/CT-DNA with the Pd1–Pd3 complexes. As the quantity of Pd1–Pd3 complexes increased, the emission intensity at 612 nm dropped, indicating competition between them. As the concentration of the Pd1–Pd3 complexes increased, the emission intensity at 612 nm decreased, indicating competition between EtBr and the investigated Pd(II) complexes for CT-DNA. The displacement of EtBr from the EtBr/CT-DNA adduct to accomplish the interaction of the Pd(II) complex with CT-DNA demonstrates that palladium(II) complexes intercalate in the DNA helix. 

The Stern–Volmer constant (K_sv_) was used to determine the degree of interaction between the Pd1–Pd3 complexes and CT-DNA (see Table 2). Based on the Ksv values, it can be concluded that the studied complexes have a strong attraction to CT-DNA and displace EtBr from the EtBr/CT-DNA adduct. The stability constant (K_a_) and the number of binding sites were determined based on the experimental data (Table 2). The obtained values for the Stern–Volmer constant (K_sv_), stability constant (K_a_), and number of binding sites (n) demonstrate that the investigated dinuclear palladium(II) complexes interact with CT-DNA by intercalating between two DNA nucleotide chains and displacing EtBr.

These findings are consistent with the Kb values acquired using UV-Vis spectroscopy. The greater values of K_sv_ and K_a_ constants for the Pd1 and Pd2 complexes indicate a stronger interaction of these complexes with CT-DNA compared to the Pd3 complex.

### 2.3. Analysis of BSA Interactions

**Figure 4:**
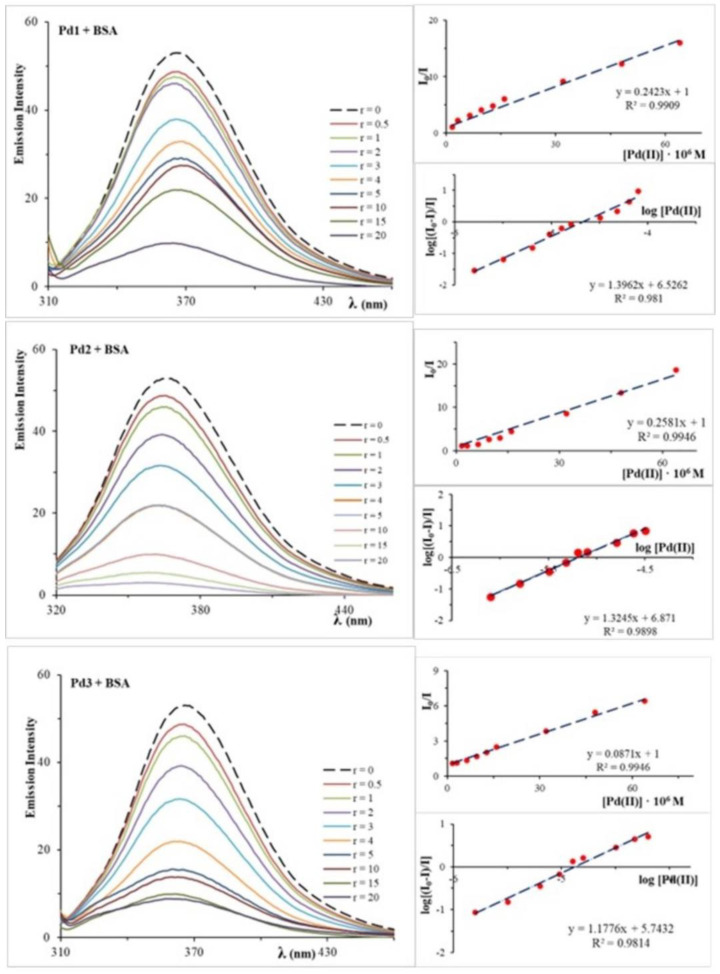
Fluorescence emission spectra of BSA in the absence and presence of Pd1–Pd3 complexes. The arrows show the intensity changes upon increasing concentrations of the complexes. Shown graphs: plot of I_0_/I versus [Q] and plot of log[(I_0_ − I)/I] versus log[Pd(II)].

Bovine serum albumin (BSA) is a heart-shaped protein with an amino acid chain composed of three homologous but structurally different domains (I, II, and III). Each domain has two sub-domains, A and B [29,30,31]. BSA is a globular, non-glycosylated plasma protein with no carbohydrate groups that transports a wide range of endogenous and exogenous substances [32]. It is well established that a drug’s affinity for plasma proteins has a direct influence on its concentration in the bloodstream and biological action. This research investigates interactions between the dinuclear Pd1–Pd3 complexes and BSA. Figure 4 depicts how the Pd1–Pd3 complexes affect the emission spectra of BSA. 

The addition of the palladium(II) complexes to a solution of BSA evoked a significant decrease in the fluorescence intensity of BSA at 365 nm, so it can be concluded that the investigated palladium(II) complexes interact with BSA molecules. In order to understand these interactions, the Stern–Volmer quenching constant (K_sv_) and quenching rate constant (k_q_) were calculated and are shown in Table 2. The palladium(II) complexes have good quenching ability of BSA fluorescence, but the Pd3 complex has a smaller K_sv_ value in comparison with the Pd1 and Pd2 complexes. The existence of a five-membered glycine and alanine ring in the Pd1 and Pd2 complexes, which has a smaller steric effect than the six-membered methionine ring in the Pd3 complex, could be a possible explanation. The palladium(II) complexes exhibit larger dynamic quenching constants than the protein’s highest rate constant (2·10^10^ M^−1^s^−1^), indicating a static quenching mechanism [33]. Furthermore, the association binding constant and number of binding sites were determined and are reported in Table 2. The Pd1–Pd3 complexes have high K_a_ constants, indicating binding to BSA. The Pd1–Pd3 complexes have a high binding constant to serum albumin, allowing efficient transport and distribution throughout the body. Palladium(II) complexes can be released once they reach their target [29,34,35]. The number of binding sites indicates the one independent class of binding sites for the investigated complexes for BSA [36]. 

### 2.4. Cytotoxicity of Pd(II) Complexes, Effect on Apoptosis, and Effect on Cell Cycle

**Figure 5:**
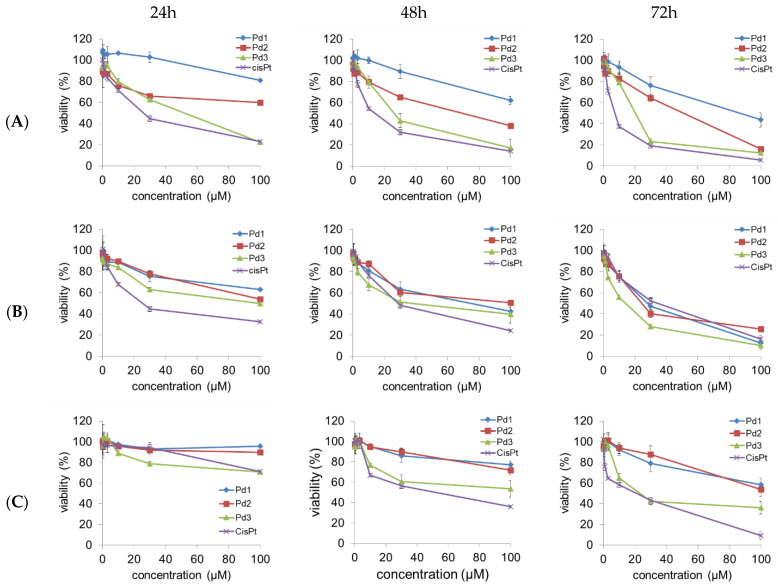
The effects of Pd(II) complexes (Pd1–Pd3) and cisplatin (CisPt) on the viability of human breast carcinoma cells with estrogen, progesterone, and glucocorticoid receptors MCF-7: (**A**) human breast carcinoma cells MDA-MB-231 and (**B**) human non-tumor cells MRC-5 (**C**).

The cytotoxicity of the three synthesized Pd(II) complexes Pd1–Pd3 was evaluated in human breast carcinoma cells with estrogen, progesterone, and glucocorticoid receptors (MCF-7), human breast carcinoma cells (MDA-MB-231), and non-cancerous human fibroblast (MRC-5) by an MTT test after 24, 48, and 72 h of treatment. The cytotoxicity of cisplatin has also been examined for comparison purposes. The results of our research undoubtedly show that all Pd(II) complexes and cisplatin show dose-dependent cytotoxic effects against MCF-7 and MDA-MB-231 cells in vitro (Figure 5). The calculated IC_50_ values for the Pd(II) complexes and cisplatin are presented in Table 3.

All tested Pd(II) complexes and cisplatin significantly decreased the viability of both MCF-7 and MDA-MB-231 cells after 24, 48, and 72 h of treatment.

The Pd3 complex displayed the highest cytotoxic activity after all exposure times and against both MCF-7 and MDA-MB-231 cell lines. In addition, the Pd3 complex showed higher cytotoxic activity against MDA-MB-231 cells after 72 h of exposure compared to cisplatin under the same conditions, as well as the complex Pd1 (Figure 5 and Table 3). All tested Pd(II) complexes had less pronounced cytotoxic effect on human healthy cells (MRC-5 fibroblasts) in comparison with cisplatin, except the Pd3 complex after exposure for 24 h. These results indicate that the newly synthetized complexes (Pd1–Pd3) have selective effects toward human breast cancer cell lines.

**Figure 6:**
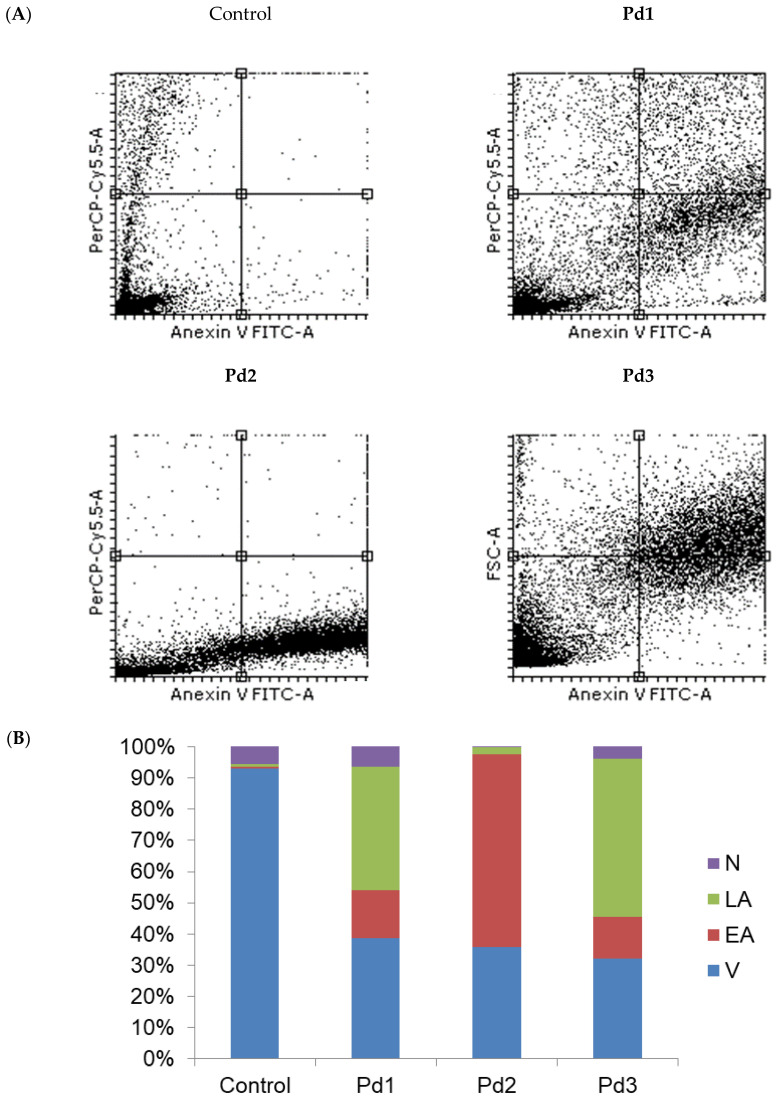
Three tested Pd(II) complexes (Pd1–Pd3) decrease viability of treated human breast carcinoma cells with estrogen, progesterone, and glucocorticoid receptor MCF-7 cells predominantly by induction of apoptosis. (**A**) Representative flow cytometry plots using annexin V-FITC/PI staining for apoptosis. (**B**) The average percentage of MCF-7 viable (V) cells, early apoptotic (EA) cells, late apoptotic (LA) cells, and necrotic (N) cells after 24 h treatment with IC_50_ of Pd1–Pd3. The control group is untreated cells.

**Figure 7:**
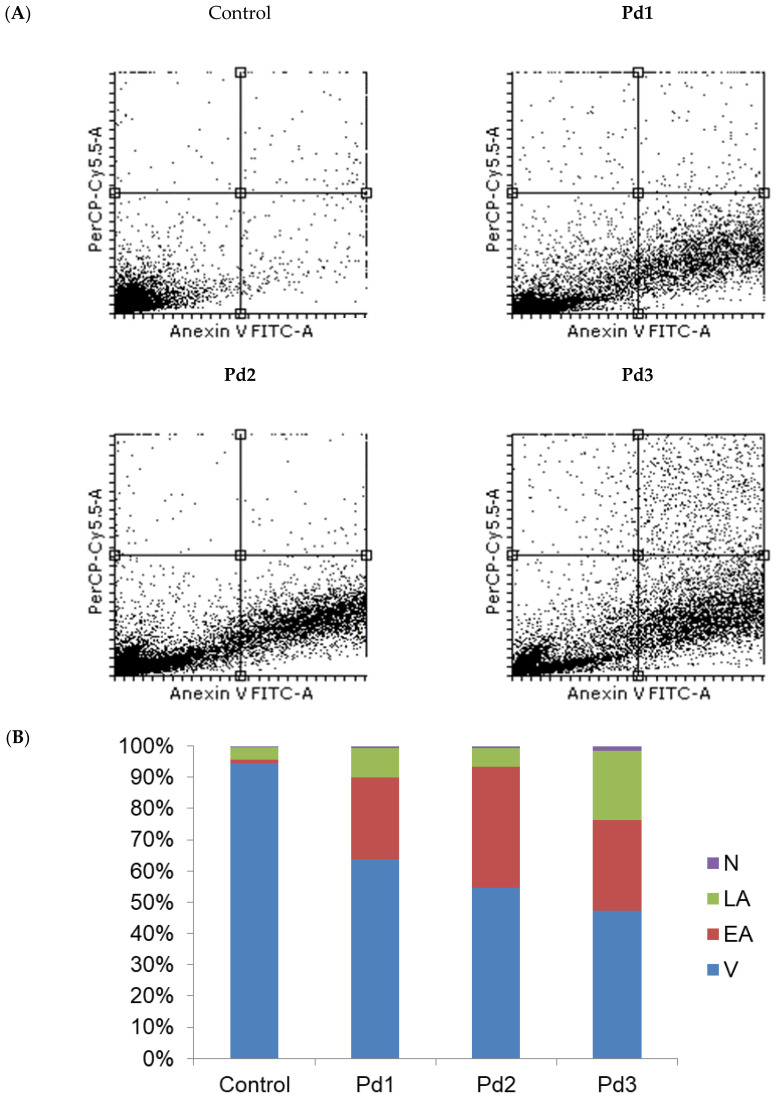
Three tested Pd(II) complexes (Pd1–Pd3) decrease viability of treated human breast carcinoma cells MDA-MB-231 predominantly by induction of apoptosis. (**A**) Representative flow cytometry plots using annexin V-FITC/PI staining for apoptosis. (**B**) The average percentage of MDA-MB-231 viable (V) cells, early apoptotic (EA) cells, late apoptotic (LA) cells, and necrotic (N) cells after 24 h treatment with IC_50_ of Pd1–Pd3. The control group is untreated cells.

**Figure 8:**
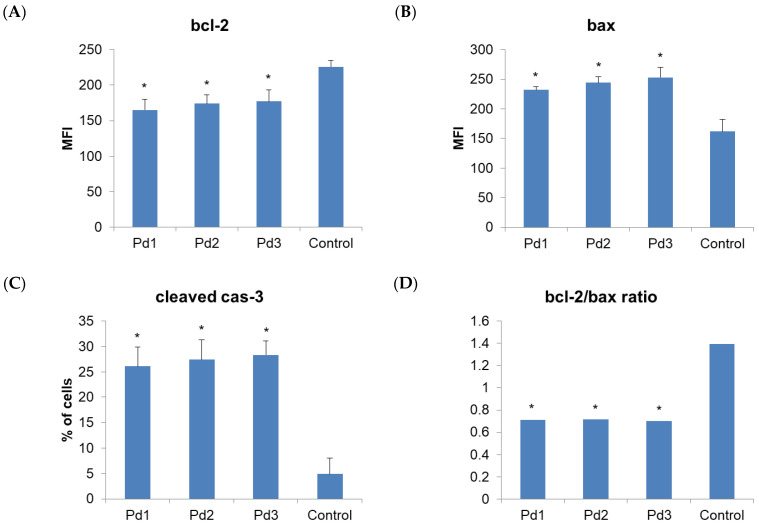
Pd(II) complexes Pd1–Pd3 induce apoptosis of human breast carcinoma cells MDA-MB-231 via a caspase-dependent pathway. (**A**) MFI values (mean fluorescence intensity) for anti-apoptotic protein bcl-2 of MDA-MB-231 cells treated with IC_50_ of Pd1–Pd3. Control group is untreated cells. (**B**) MFI values for pro-apoptotic protein active BAX of MDA-MB-231 cells treated with IC_50_ of Pd1–Pd3. Control group is untreated cells. (**C**) The percentages of cells displaying fluorescence for active (cleaved) caspase-3. (**D**) Bcl-2/BAX ratio for MDA-MB-231 cells. Results are presented as mean ± standard deviation. * *p* < 0.05 compared to control cells.

**Figure 9:**
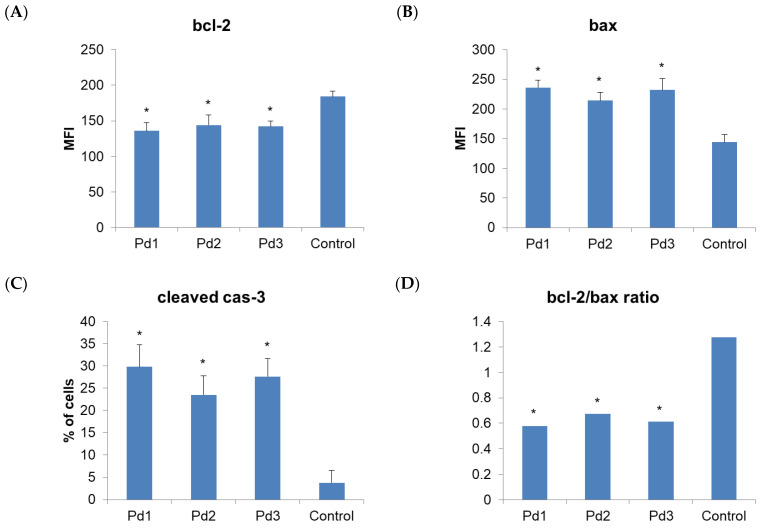
Pd(II) complexes Pd1–Pd3 induce apoptosis of human breast carcinoma cells with estrogen, progesterone, and glucocorticoid receptors MCF-7 via a caspase-dependent pathway. (**A**) MFI values (mean fluorescence intensity) for anti-apoptotic protein bcl-2 of MCF-7 cells treated with IC_50_ of Pd1–Pd3. Control group is untreated cells. (**B**) MFI values for pro-apoptotic protein active BAX of MCF-7 cells treated with IC_50_ of Pd1–Pd3. Control group is untreated cells. (**C**) The percentages of cells displaying fluorescence for active (cleaved) caspase-3. (**D**) Bcl-2/BAX ratio for MCF-7 cells. Results are presented as mean ± standard deviation. * *p* < 0.05 compared to control cells.

Therefore, the next step of our research was to examine the type of MCF-7 and MDA-MB-231 cell death induced by these complexes. An annexin V/PI staining assay was performed for that purpose. The results show that apoptosis was induced by all the tested Pd(II) complexes (Figure 6). In the case of MCF-7 cells, the highest percentage of necrotic cells (6.3%) was observed after treatment with an IC_50_ dose of the Pd1 complex. When the MDA-MB-231 cells were observed, the highest percentage of necrotic cells was detected after treatment with the complex Pd3 (Figure 7). The next step was to investigate whether the Pd(II) complexes affected the cytoplasmic concentration of the antiapoptotic protein bcl-2 and proapoptotic protein BAX. Also, activation of the caspase-3 was evaluated in MCF-7 and MDA-MB-231 cancer cells treated with the Pd(II) complexes. The results show that all the tested Pd(II) complexes significantly reduced the amount of antiapoptotic protein bcl-2 in both MCF-7 and MDA-MB-231 cancer cells (Figure 8 and Figure 9, *p* < 0.05). There was also a significant increase in the concentration of proapoptotic protein BAX in the MCF-7 and MDA-MB-231 cancer cells treated with an IC_50_ dose of the Pd(II) complexes compared to untreated cells (Figure 8 and Figure 9, *p* < 0.05). The percentage of cells containing active caspase-3 was also increased in tumor cells treated by the Pd(II) complexes compared to untreated cells (Figure 8 and Figure 9, *p* < 0.05). Therefore, the Pd(II) complexes Pd1–Pd3 decreased the bcl-2/BAX ratio in comparison to untreated cells, which led to the activation of caspase-3 and induction of apoptosis. Since the discovery of platinum-based drugs, many new metal complexes have been synthesized and evaluated to discover potential anticancer drugs.

We synthesized three new Pd(II) complexes, Pd1–Pd3, and investigated their cytotoxicity and mechanism of action against human breast carcinoma cells with estrogen, progesterone, and glucocorticoid receptors (MCF-7), human breast carcinoma cells (MDA-MB-231), and one non-cancerous type of human cells, MRC-5 cells. We discovered that all three Pd(II) complexes had a strong and selective cytotoxic effect on both tested breast carcinoma cells. Also, the Pd3 complex showed as more efficient on MDA-MB-231 cells after 72 h compared to cisplatin. The effect of the Pd1 and Pd2 complexes is comparable when looking at the effect on MCF-7 cells, while the effect of the Pd2 complex is slightly more pronounced on MDA-MB-231 cells compared to the Pd1 complex. In the context of the selectivity of the newly synthesized palladium complexes, it was shown that the complexes Pd1 and Pd2 have a pronounced selective effect against human breast cancer cell lines. The selectivity of the complex Pd3 is slightly lower but still better compared to cisplatin.

Pd(II) complexes exhibit significant anticancer potential against MCF-7 human breast cancer cells, primarily through the induction of apoptosis [37]. This effect and mechanism were also confirmed in our study, where, after treatment with the Pd complexes, the largest number of cells transitioned from viable to early and late apoptosis stages. These complexes demonstrate cytotoxic and antiproliferative properties, disrupting cellular metabolism and inducing cell death [37,38]. Pd(II) complexes demonstrate both short-term and long-term antiproliferative effects, significantly reducing cell growth and abolishing long-term proliferation. They also inhibit cell migration and adhesion, induce morphological alterations, and cause cellular shrinkage. The complexes interact with DNA, inducing the cleavage of double-stranded DNA, which accounts for their cytotoxic effects [38,39].

Studies have shown that Pd(II) complexes can trigger apoptosis via cell death receptors and the production of reactive oxygen species, with MCF-7 cells showing a more pronounced apoptotic response compared to other cell lines like MDA-MB-231 [37]. Specific Pd(II) complexes, such as those with picolinic acid, have demonstrated potent activity against MCF-7 cells. Furthermore, the metabolic response induced by these complexes can be faster than that of cisplatin, potentially leading to quicker patient recovery [40].

Palladium(II) complexes have demonstrated cytotoxic and growth-inhibitory effects on the MDA-MB-231 human breast adenocarcinoma cell line [41,42,43,44,45]. These complexes induce a range of cellular changes, including decreased cell number and morphological alterations, which are indicative of cytotoxicity. The cytotoxic effect may be due to the interaction of palladium(II) complexes with DNA, leading to the formation of DNA adducts [41,42,43]. One study showed that the complexation of thiosemicarbazone to Pd(II) may be a viable strategy for developing antitumor agents. Furthermore, Pd(II) complexes can alter the expression levels of various proteins in MDA-MB-231 cells [45]. Other metal complexes also demonstrated potent inhibitory effects against the MDA-MB-231 cell line [42,44].

## 3. Materials and Methods

### 3.1. Chemicals and Equipment

Commercially pure chemicals, including K_2_[PdCl_4_], glycine (Gly), alanine (Ala), methionine (Met), pyrazine (pz), deoxyribonucleic acid from calf thymus (CT-DNA), phosphate-buffered saline (PBS, 10 mM, pH 7.40, containing 2.70 mM KCl and 137 mM NaCl), ethidium bromide (3,8-diamino-5-ethyl-6-phenylphenanthridiniumbromide (EtBr)), and bovine serum albumin (BSA) were purchased from the Sigma-Aldrich Chemical Co. and used as received. All the other common chemicals were of reagent grade and used without purification. The mononuclear palladium(II) complexes K[Pd(Gly-*N*,*O*)Cl_2_], K[Pd(Ala-*N*,*O*)Cl_2_]·2H_2_O and [Pd(Met-*S*,*N*)Cl_2_] were synthesized using a method reported in the literature [46,47]. All pH measurements were made at 298 K. The pH meter (S220 Seven Compact, pH/Ion, Mettler Toledo) was calibrated with a buffer solution of pH 4.00 and 7.00. The purity of the complexes was checked by elemental microanalyses, ^1^H NMR, and UV-Vis spectroscopy. Elemental analyses for C, H, and N were performed by standard micro-methods using an ELEMENTAR Vario ELIII C.H.N.S.O analyzer. The ^1^H NMR spectra were acquired on a Varian Gemini 2000 (^1^H at 200 MHz) at 298 K in D_2_O. All chemical shifts are referenced to TSP (trimethylsilylpropanoic acid). The UV-Vis spectra were acquired by dissolving the appropriate palladium(II) complex in water (0.05 mM) in the wavelength range of 200–500 nm on a Shimadzu double-beam spectrophotometer fitted with thermostated 1.00 cm quartz Suprasil cells. CT-DNA stock solution was made in 10 mM PBS buffer and stored in a refrigerator at 277 K, providing a UV absorbance ratio at 260 and 280 nm (A_260_/A_280_) of around 1.8–1.9, indicating that the CT-DNA was sufficiently free of protein contamination. The CT-DNA concentration was measured by UV absorbance at 260 nm (ε = 6600 M^−1^cm^−1^) [48,49]. The BSA solution was prepared based on its molecular weight of 66 463 in 10 mM PBS buffer and kept in a refrigerator at 277 K for no longer than seven days [5].

### 3.2. Synthesis of [{Pd(Gly-N,O)Cl}_2_(µ-pz)], [{Pd(Ala-N,O)Cl}_2_(µ-pz)], and [{Pd(Met-S,N)Cl}_2_(µ-pz)](NO_3_)_2_ Complexes

The complexes [{Pd(Gly-*N,O*)Cl}_2_(*µ*-pz)] (Pd1), [{Pd(Ala-*N,O*)Cl}_2_(*µ*-pz)] (Pd2), and [{Pd(Met-*S,N*)Cl}_2_(*µ*-pz)](NO_3_)_2_ (Pd3) were prepared by the modification of the procedure from the literature [5,50,51,52,53,54]. In a suspension of the mononuclear K[Pd(Gly-*N,O*)Cl_2_], K[Pd(Ala-*N,O*)Cl_2_]·2H_2_O, or [Pd(Met-*S,N*)Cl_2_] complex (0.343 mmol) in 10 mL of dimethylformamide (dmf), a dimethylformamide solution of AgNO_3_ (57.20 mg; 0.337 mmol) was added. The reaction mixture was stirred at room temperature and kept in the dark overnight. The precipitated AgCl was separated by straining, and the pale yellow solution of the palladium(II) complex ([Pd(Gly-*N,O*)Cl(dmf)], [Pd(Ala-*N,O*)Cl(dmf)], and [Pd(Met-*S,N*)Cl(dmf)]^+^) in dimethylformamide was used as a starting substance for the synthesis of the dinuclear [{Pd(Gly-*N,O*)Cl}_2_(*µ*-pz)], [{Pd(Ala-*N,O*)Cl}_2_(*µ*-pz)], and [{Pd(Met-*S,N*)Cl}_2_(*µ*-pz)](NO_3_)_2_ complexes. In the solution containing the [Pd(Gly-*N,O*)Cl(dmf)], [Pd(Ala-*N,O*)Cl(dmf)] or [Pd(Met-*S,N*)Cl(dmf)]^+^ complex, the solution obtained by dissolving pyrazine (pz) in 5 mL was slowly added in a molar ratio of 2:1. The reaction mixture was left in the dark while stirring at 298 K for about 4 h. On a rotary vacuum evaporator, the volume of the solution was reduced. After adding dichloromethane, a light yellow precipitate of [{Pd(Gly-*N,O*)Cl}_2_(*µ*-pz)], [{Pd(Ala-*N,O*)Cl}_2_(*µ*-pz)], or [{Pd(Met-*S,N*)Cl}_2_(*µ*-pz)](NO_3_)_2_ was obtained. The precipitate from each complex was filtered, washed with methanol, and air dried. The purity and content of the complex were determined by elemental microanalysis, ^1^H NMR, and UV-Vis spectroscopy.

[{Pd(Gly-*N,O*)Cl}_2_(*µ*-pz)] (Pd1). Yield: 68% (59.70 mg). Anal. Calcd. For Pd1 (C_8_H_12_N_4_Cl_2_O_4_Pd_2_: FW = 511.95): C, 18.77; H, 2.36; N, 10.94%. Found: C, 18.56; H, 2.48; N, 10.79%. ^1^H NMR (200 MHz, D_2_O, δ, ppm): 3.52 (s, CH_2_), 8.83 (d, CH-pz). UV–Vis (H_2_O, λ_max_, nm): 262(ε = 1.03·10^3^ M^−1^ cm^−1^). IR (KBr, ν, cm^−1^): 3295–3102 (N-H stretch); 1686 (COO); and 1573–1378 (C=N/C=C).

[{Pd(Ala-*N,O*)Cl}_2_(*µ*-pz)] (Pd2). Yield: 72% (66.68 mg). Anal. Calcd. For Pd2 (C_10_H_16_N_4_Cl_2_O_4_Pd_2_: FW = 540.00): C, 22.24; H, 2.29; N, 10.38%. Found: C, 22.11; H, 2.21; N, 10.49%. ^1^H NMR (200 MHz, D_2_O, δ, ppm): 1.46 (d, CH_3_), 3.69 (q, CH), 8.92 (s, CH-pz). UV–Vis (H_2_O, λ_max_, nm): 260 (ε = 1.06·10^3^ M^−1^ cm^−1^). IR (KBr, ν, cm^−1^): 3276–3054 (N-H stretch); 1687 (COO); and 1572–1364 (C=N/C=C).

[{Pd(Met-*S,N*)Cl}_2_(*µ*-pz)](NO_3_)_2_ (Pd3). Yield: 58% (78.21 mg). Anal. Calcd. For Pd3 (=(C_14_H_26_N_6_Cl_2_O_10_S_2_Pd_2_ (Mr = 786.27): C, 21.39; H, 3.30; N, 10.69%. Found: C, 21.45; H, 3.55; N, 10.74%. ^1^H NMR (200 MHz, D_2_O, δ, ppm): 2.58 (s, S-CH_3_), 2.23 (m, CH_2_-β), 2.73 (m, CH_2_-γ), 3.94 (m, CH), 8.89 (s, CH-pz). UV–Vis (H_2_O, λ_max_, nm): 259 (ε = 2.17·10^3^ M^−1^ cm^−1^). IR (KBr, ν, cm^−1^): 3310–3128 (O-H and N-H stretch); 1723 (COOH); and 1632–11,592 (C=N/C=C).

### 3.3. Analysis of DNA Binding and Interactions

#### 3.3.1. Absorption Spectroscopic Measurements

The interactions between the Pd1–Pd3 complexes and deoxyribonucleic acid isolated from calf thymus (CT-DNA) were investigated using UV-Vis spectroscopy. The solutions used for UV-Vis measurements were prepared in 10 mM phosphate buffer solution (pH = 7.40). The absorbance values were recorded at 310 K after each successive addition of CT-DNA solution and equilibration (4 h). To determine the intrinsic binding constant (K_b_) of CT-DNA molecules to the palladium(II) complexes, the UV-Vis spectra of solutions obtained by mixing the complexes with CT-DNA solution were recorded. In all solutions, the complex concentration was constant (0.0152 mM), while the CT-DNA concentration was in the interval (0–0.0243) mM ([Pd(II)]/[CT-DNA] = 0.0–1.6). The intrinsic binding constants (K_b_) were determined according to the equation: [DNA]/(ε_a_ − ε_f_) = [DNA]/(ε_b_ − ε_f_) + 1/K_b_·(ε_b_ − ε_f_) [55,56], where [DNA] is the concentration of CT-DNA, ε_a_ is the extinction coefficient of the complex at a given CT-DNA concentration, and ε_f_ and ε_b_ are the extinction coefficients of the complex in free solution and when it is fully bound to CT-DNA, respectively. The obtained results are shown graphically as a dependence of [DNA]/(ε_a_ − ε_f_) on [DNA]. The slope of the resulting line has a value of 1/(ε_b_ − ε_f_), while the intercept on the y-axis is 1/K_b_·(ε_b_ − ε_f_). The value of K_b_ was calculated from the ratio of the slope of the intercept. The free energy (ΔG) of the Pd(II)/CT-DNA complexes was estimated using the following equation: ΔG = RTlnK_b_, where R is the ideal gas constant, T = 310 K, and K_b_ represents the intrinsic binding constant.

#### 3.3.2. Competitive Fluorescence Measurements

Using emission fluorescence spectroscopy, the interactions of the Pd1–Pd3 complexes with CT-DNA in the presence of ethidium bromide (EtBr) were examined. The EtBr/CT-DNA complex was initially prepared by mixing EtBr and CT–DNA in 1:1 M ratio with concentrations of 0.0185 mM in 10 mM PBS at pH = 7.40 at 298 K and analyzed by fluorescence measurement. Then, gradually increasing concentrations of the Pd1–Pd3 complexes (0–0.0167 mM) were successively added, and the change in the fluorescence intensity was measured. Emission spectra were recorded in the range of 550–750 nm, with excitation at 527 nm and fluorescence emission at 612 nm. Before taking measurements, each system was shaken and incubated for 5 min at 298 K. The palladium(II) complexes did not show fluorescence under these conditions. The Stern–Volmer constant (K_sv_) was determined based on the equation [57]: I_0_/I = 1 + K_sv_[Pd(II)] = 1 +k_q_τ_0_[Pd(II)], where I_0_ and I are the fluorescence intensities before and after adding the Pd(II) complex to the EtBr/CT-DNA solution, [Pd(II)] is the total concentration of the palladium(II) complex, K_sv_ is the Stern–Volmer quenching constant, k_q_ is quenching rate constant, and τ_0_ is the average lifetime of the biomolecule without a quencher. The results are graphically presented as the dependence of I_0_/I on [Pd(II)]. The Stern–Volmer constant (K_sv_) was determined from the slope of the obtained line. The stability constant (K_a_) and the number of binding sites (n) were determined based on the Scatchard equation [58]: log (I_0_ − I)/I = logK_a_ + n·log[Pd(II)], where I_0_ and I are the fluorescence intensities before and after adding the palladium(II) complex to the EtBr/CT-DNA solution, [Pd(II)] is the concentration of the palladium(II) complex, and n is number of binding sites. The results are graphically presented as the dependence of log(I_0_ − I)/I on log[Pd(II)]. The intersection of the line with the y-axis provided the value of K_a_, and the slope of the line determined the number of binding sites (n).

### 3.4. Analysis of BSA Interactions

The interaction between BSA and Pd1–Pd3 was studied using steady-state fluorescence emission measurements at 298 K in 10 mM PBS buffer (pH 7.40). The Pd1–Pd3 complexes were used as quenchers to measure the emission intensity of tryptophan residues in BSA (0.0016 mM) at 352 nm. The concentration was increased up to 0.04 mM. Fluorescence spectra were obtained in the 300–500 nm region with an excitation wavelength of 295 nm. The fluorescence spectra of substances in buffered solutions were recorded under identical experimental conditions, and no fluorescence emission was observed. The fluorescence quenching is calculated by the Stern–Volmer equation previously described in the Materials and Methods section of this paper (Section 3.3.2). In this case, τ_0_ = 1.0·10^−8^ s for BSA was used [59]. The binding constant was calculated by the Scatchard equation: log[(I_0_−I)/I] = logK_a_ + n⋅log[Pd(II)], where K_a_ is the association binding constant, and n is the number of binding sites that have been obtained from the plot of log[(I_0_ − I)/I] versus log[Pd(II)].

### 3.5. Cytotoxicity Assay

#### 3.5.1. Cell Cultures

In this study, two experimental groups of cancer cell lines were utilized: human breast carcinoma cells with estrogen, progesterone, and glucocorticoid receptors (MCF-7 and HTB-22^™^) and human breast carcinoma cells (MDA-MB-231 and HTB-26^™^). The control group consisted of non-cancerous human fibroblasts (MRC-5 and CCL-171^™^). All cell lines employed in the research were sourced from the American Type Culture Collection (ATCC, Manassas, VA, USA). The cells were cultured in a complete medium comprising high-glucose DMEM supplemented with 10% fetal bovine serum and 200 mM L-glutamine (reagents supplied by Sigma-Aldrich, St. Louis, MO, USA). Cell cultivation was carried out in 25 cm^2^ flasks (Thermo Fisher Scientific, Waltham, MA, USA) at 37 °C under conditions of absolute humidity and a 5% CO_2_ atmosphere.

#### 3.5.2. MTT Assay

The cytotoxic effects of two newly synthesized Pd(II) complexes were evaluated on MCF-7, MDA-MB-231, and MRC-5 cells using the MTT assay [60]. Additionally, the cytotoxicity of cisplatin, due to its established clinical relevance, was assessed across all cell lines. Cells were harvested during the exponential growth phase, counted, and seeded at a density of 5 × 10^3^ cells per well in 96-well culture plates. Following an initial incubation at 37 °C in a 5% CO_2_ atmosphere for 24 h, the cells were treated with varying concentrations of Pd(II) complexes and cisplatin (0.3, 1, 3, 10, 30, and 100 μM) alongside a control group cultured in a complete medium. The cells were maintained under identical incubation conditions (37 °C, 5% CO_2_, absolute humidity) for 24, 48, and 72 h. After incubation, the medium was removed, and an MTT solution was added to each well. Cells were further incubated for 2 h to allow the reduction of thiazolyl blue tetrazolium bromide in the MTT solution. Subsequently, the MTT solution was carefully removed, and the resulting formazan crystals were dissolved in DMSO. The plates were shaken in the dark for 10 min, and the absorbance of the purple-colored solution was measured at 595 nm using a microplate reader (Zenyth 3100, Anthos Labtec Instruments, Salzburg, Austria).

All experiments were conducted in triplicate and repeated in three independent runs. Cell viability was calculated as a percentage by dividing the absorbance of treated cells (minus the blank absorbance) by the average absorbance of untreated control cells (minus the blank absorbance), then multiplying by 100:

% of the viable cells = ((absorbance of treated cell-absorbance of blank)/(absorbance of untreated cell-absorbance of blank)) × 100

The IC_50_ values (the concentration required to reduce cell viability by 50% relative to the control) were determined by fitting the logarithm-transformed dose–response data obtained from the MTT assay using Microsoft Office Excel 2010.

#### 3.5.3. Annexin V/7AAD Assay

The type of cell death induced by the Pd(II) complexes Pd1–Pd3 was estimated by an annexin V–fluorescein isothiocyanate (FITC)/propidium iodide (PI) Apoptosis Kit (BD Biosciences, Franklin Lakes, NJ, USA). MCF-7, MDA-MB-231, and MRC-5 cells were incubated with appropriate IC_50_ concentrations of the Pd1–Pd3 complexes or with media alone (control) for 24 h at 37 °C in an atmosphere of 5% CO_2_ and absolute humidity. Then, MCF-7, MDA-MB-231, and MRC-5 cells were trypsinized, washed in phosphate buffer saline (PBS), centrifuged, and resuspended in 100 μL of ice-cold binding buffer. In addition, cells were stained with both 10 μL of annexin V-FITC and 20 μL of PI, incubated for 15 min in the dark at room temperature, and then to each tube, 400 μL of binding buffer was added. We measured samples using the flow cytometer Cytomics FC500 (Beckman Coulter, Brea, CA, USA). The data obtained were also analyzed using FlowJo V10 Software. The measurements are presented as density plots of annexin V-FITC and PI staining.

#### 3.5.4. Assessment of Apoptosis

Our research aimed to examine the expression of the proapoptotic protein BAX, antiapoptotic protein bcl-2, and the percentage of cells containing active caspase-3. MCF-7 and MDA-MB-231 cells were incubated for 24 h with an IC_50_ concentration of the Pd1–Pd3 complexes or in a complete cell culture medium (control). In addition, MCF-7 and MDA-MB-231 cells were washed three times with ice-cold PBS, resuspended, fixed, and permeabilized (Fixation and Permeabilization Kit, eBioscience, San Diego, CA, USA). For bcl-2 staining, the cells were incubated with 1:1000 bcl-2 fluorescein isothiocyanate (FITC) primary antibody (mhbcl01, Life technologies, Thermo Fisher Scientific, Waltham, MA, USA) for 15 min at room temperature. Additional staining included incubation of permeabilized MCF-7 and MDA-MB-231 cells for 30 min with 1:1000 of primary antibodies for active BAX (N20, sc-493; Santa Cruz Biotech Inc., Dallas, TX, USA) and cleaved caspase-3 (#9661, Cell signaling Technology, Danvers, MA, USA). Also, cells were washed with PBS and incubated with the 1:2000 secondary goat anti-rabbit IgG-FITC antibody (Ab6717-1, Abcam, Cambridge Biomedical Campus, Cambridge, UK) for 30 min. Afterward, cells were washed in PBS and analyzed by flow cytometry. The fluorescence of at least 15,000 events/sample was measured using an FC500 (Beckman Coulter, Brea, CA, USA). Fluorescence intensity was standardized using isotype-matched negative control antibodies. The mean fluorescence intensities for BAX and bcl-2 (MFIs) were calculated as the ratio of the raw mean channel fluorescence to isotype control levels, respectively, and represented the expression level of these proteins. The cleaved caspase-3 concentrations were evaluated as the percentages of cells displaying fluorescence [61].

## 4. Conclusions

Using UV-Vis spectroscopy and fluorescence spectroscopy, the interactions of synthesized palladium(II) dinuclear complexes with deoxyribonucleic acid were investigated. The results of the experiments show that the tested palladium(II) complexes bind to CT-DNA and displace the ethidium–bromide intercalator from the CT-DNA molecules and act as intercalators. The higher numerical value of K_b_ and K_sv_ for the Pd1 and Pd2 complexes compared to Pd3 can be attributed to the smaller steric effect of the bidentate-coordinated glycine and alanine, which build a five-membered chelate ring, compared to methionine, which builds six-membered chelate fingers through bidentate coordination to the Pd(II) ion. The most pronounced antitumor activity was shown by the Pd3 complex with methionine as a ligand. This complex showed greater activity against the MDA-MB-231 cell line after 72 h of exposure compared to cisplatin. The main mechanism of action was the induction of apoptosis through the activation of caspase-3. Research is needed to examine in more detail other possible molecular mechanisms of action, as well as the pharmacokinetics and potential toxicity of the resulting complexes.

The results obtained in this paper contribute to a better understanding of the interactions of the dinuclear complexes of palladium(II), which contain pyrazine as a bridging ligand, with biologically important molecules, such as DNA and BSA, as well as the presence of cytotoxic activity and its basic mechanism.

## Figures and Tables

**Table 1:** ^1^H NMR chemical shifts (δ, ppm) and multiplicities for ligands glycine (Gly), alanine (L-Ala), methionine (L-Met), pyrazine (pz), and palladium(II) complexes in D_2_O.

Ligand/Complex	^1^H NMR (ppm)
Gly	3.85 (s, CH_2_)
Pd1	3.52 (s, CH_2_); 8.83 (d, CH-pz)
L-Ala	1.40 (d, CH_3_) 3.55 (q, CH)
Pd2	1.46 (d, CH_3_); 3.69 (q, CH); 8.92 (s, CH-pz)
L-Met	2.14 (s, S-CH_3_); 2.17 (m, CH_2_-β); 2.65 (t, CH_2_-γ); 3.87 (m, CH)
Pd3	2.58 (s, S-CH_3_); 2.23 (m, CH_2_-β); 2.73 (m, CH_2_-γ); 3.94 (m, CH); 8.89 (s, CH-pz)
pz	8.66 (s)

**Table 2:** The intrinsic binding constants (K_b_), Gibbs energy (ΔG), the Stern–Volmer constant (K_sv_), stability constant (K_a_), and the number of binding sites (n) of Pd1–Pd3 complexes with CT–DNA; Stern–Volmer constants (K_sv_), quenching constants (k_q_), binding constants (K_a_), and number of binding sites (n) for interactions of Pd1–Pd3 complexes with BSA.

			Pd1	Pd2	Pd3
**CT-DNA interactions**	UV-Vis	K_b_ (⋅10^4^ M^−1^)	6.33	5.31	0.96
		ΔG_298_ (kJ/mol)	−28.49	−28.04	−23.63
	Fluorescence measurements	K_sv_ (⋅10^4^ M^−1^)	12.03	17.60	5.36
		K_a_ (⋅10^4^ M^−1^)	14.40	15.20	1.48
		n	≈1	≈1	≈0.9
**BSA interactions**	Fluorescence measurements	K_sv_ (⋅10^5^ M^−1^)	2.42	2.58	0.87
		K_a_ (⋅10^6^ M^−1^)	3.36	7.43	0.56
		k_q_ (⋅10^13^ M^−1^ s^−1^)	2.42	2.58	0.87
		n	≈1.4	≈1.3	≈1.2

**Table 3:** IC_50_ values in µM for Pd(II) complexes Pd1–Pd3 and cisplatin after 24, 48, and 72 h drug exposure. Results are presented as mean ± SD and determined from the results of MTT assay in three independent experiments.

Cell Line	Time	Pd1	Pd2	Pd3	CP
MCF-7	24 h	220.2 ± 22.8	>250	58.2 ± 4.8	52.5 ± 5.9
	48 h	129.1 ± 14.8	72.7 ± 7.9	28.3 ± 2.6	12.4 ± 1.1
	72 h	86.2 ± 7.3	54.7 ± 5.1	16.1 ± 1.8	5.8 ± 0.5
MDA-MB-231	24 h	119.2 ± 12.8	103.7 ± 8.5	98.7 ± 7.9	28.3 ± 3.1
	48 h	83.1 ± 7.5	90.4 ± 7.7	29.4 ± 2.7	24.8 ± 2.1
	72 h	21.9 ± 2.4	27.6 ± 3.2	8.9 ± 0.7	20.1 ± 1.9
MRC-5	24 h	>250	>250	48.4 ± 4.2	183.7 ± 21.9
	48 h	210.4 ± 19.1	179.3 ± 19.6	93.7 ± 11.2	43.1 ± 3.6
	72 h	117.9 ± 12.1	110.1 ± 11.6	43.1 ± 3.6	19.7 ± 2.1

## Data Availability

The data supporting the findings of this study can be obtained from the corresponding author upon request.

## Supplementary Materials

The following supporting information can be downloaded at: https://www.mdpi.com/article/10.3390/molecules30071534/s1, Figure S1: ^1^H NMR spectrum of Pd1–Pd3 (200 MHz, D_2_O, 298 K); Figure S2: UV–Vis spectra of Pd1–Pd3 complexes measured in 0.05 mM water solution; Figure S3: IR spectrum of Pd1–Pd3 complexes (KBr pellet, 298 K).
