# Supplementary material for: DNA/BSA Binding Affinity and Cytotoxicity of Dinuclear Palladium(II) Complexes with Amino Acids as Ligands
Source: Molecules. 2025 Mar 30;30(7):1534. doi: 10.3390/molecules30071534 (PMC11990606; doi:10.3390/molecules30071534)
Supplement: Supplementary file 1 [file molecules-30-01534-s001.zip › molecules-3525166-supplementary.pdf]

Article

DNA/BSA Binding Affinity and Cytotoxicity of Dinuclear Palladium(II) Complexes with Amino Acids as Ligands

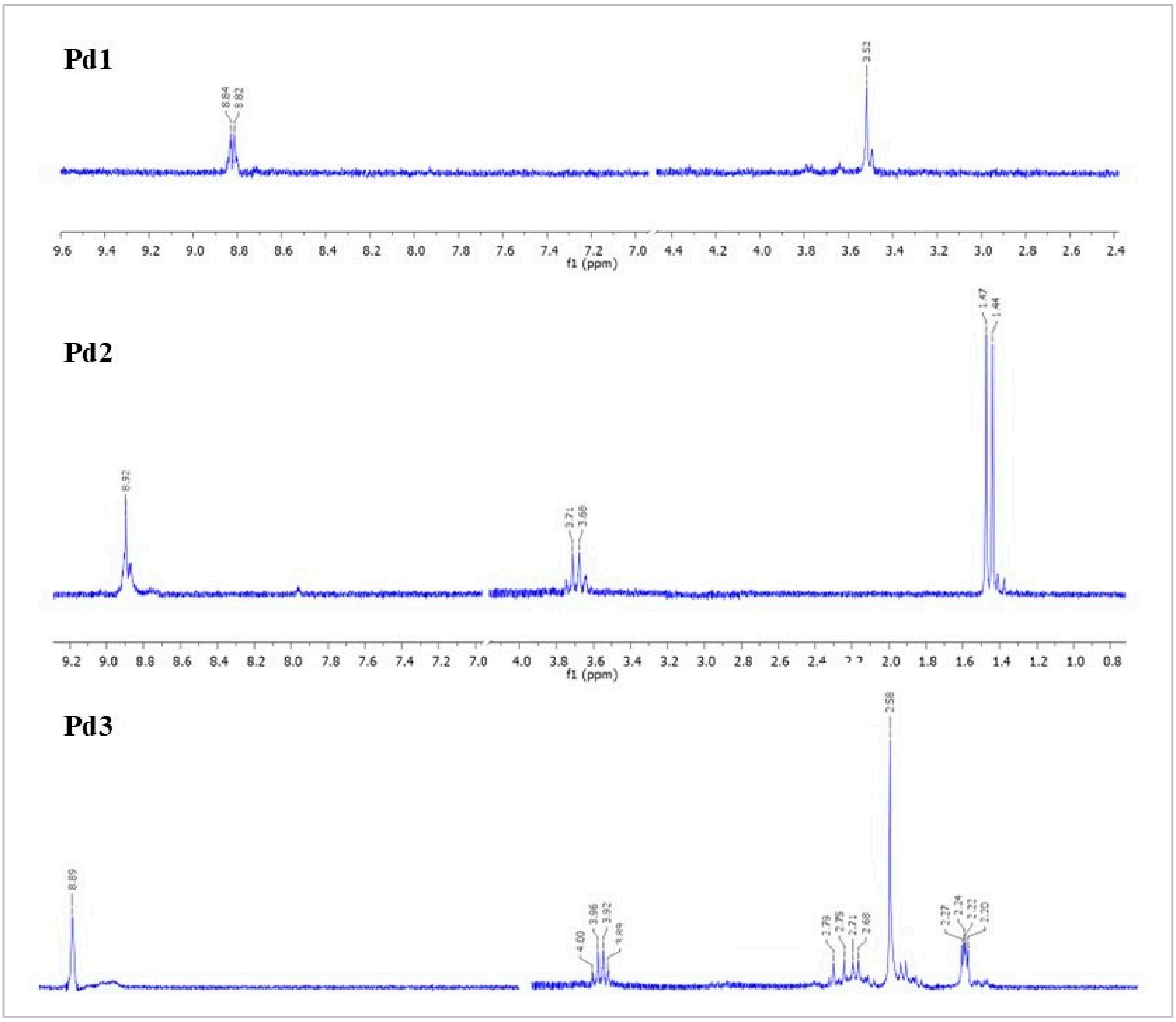

Fig. S1 ^{1}H NMR spectrum of Pd1–Pd3 (200 MHz, D_2O, 298 K).

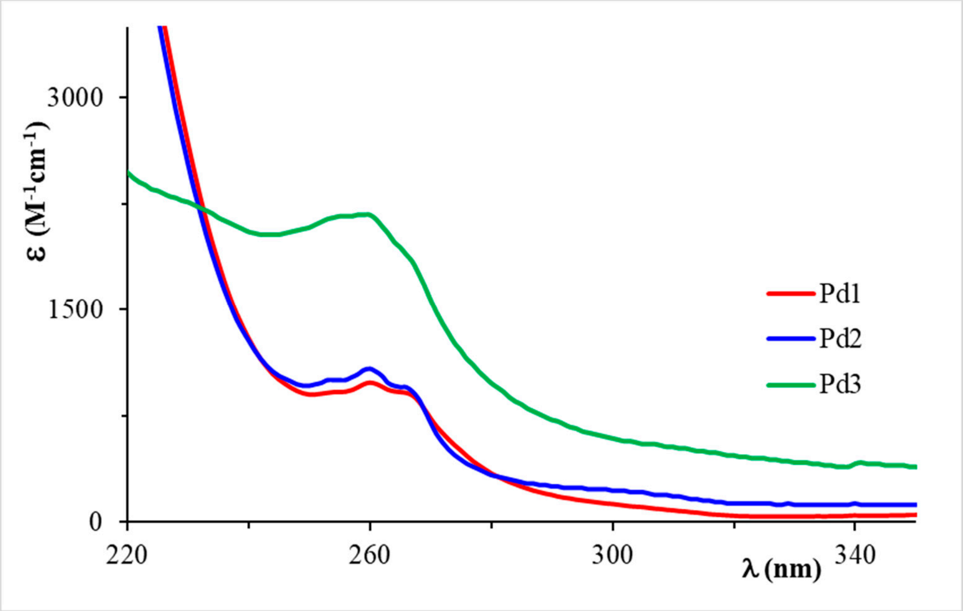

Fig. S2. UV−Vis spectra of Pd1−Pd3 complexes measured in 0.05 mM water solution.

[{Pd(Gly-N,O)Cl}$_2$(μ-pz)] (**Pd1**)

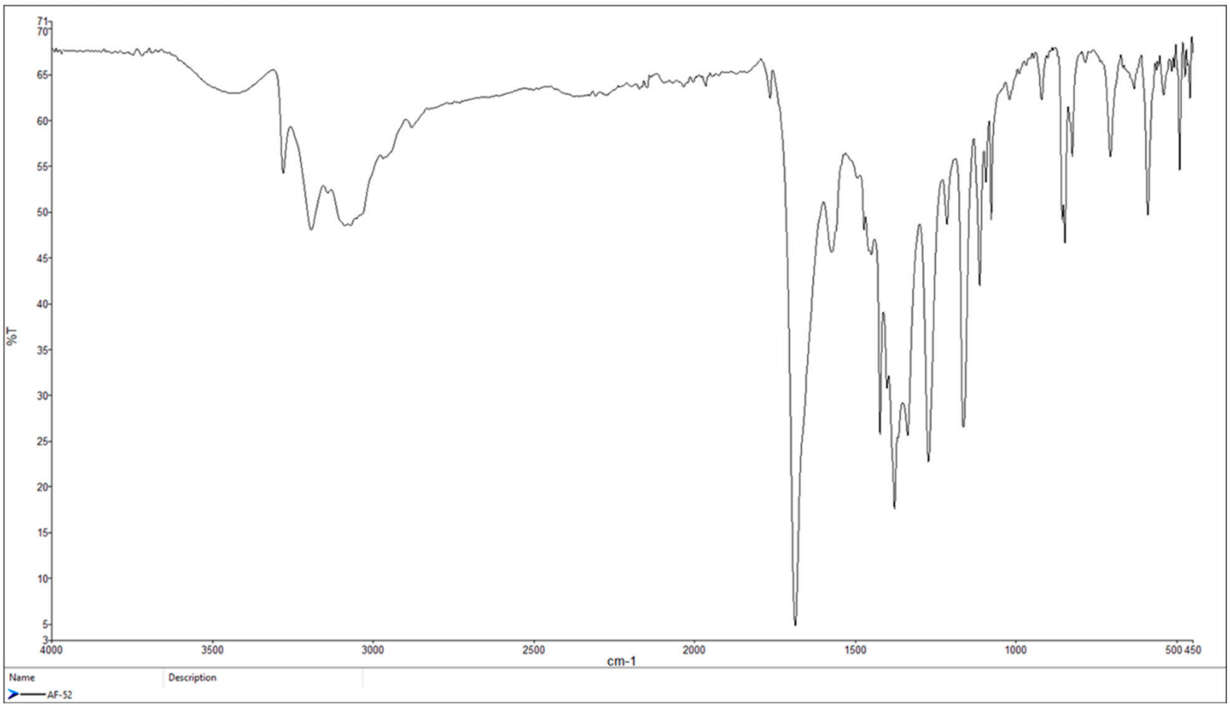

[{Pd(Ala-N,O)Cl}$_2$(μ-pz)] (**Pd2**)

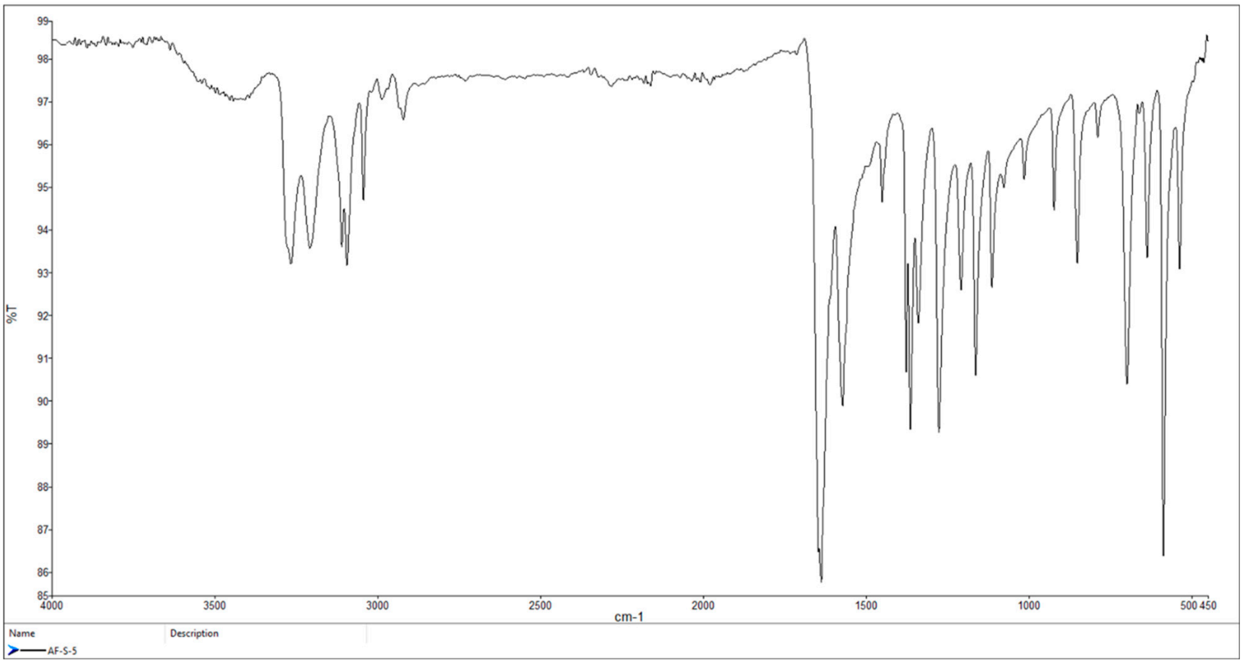

[{Pd(Met-S,N)Cl}$_2$(μ-pz)](NO$_3$)$_2$ (**Pd3**)

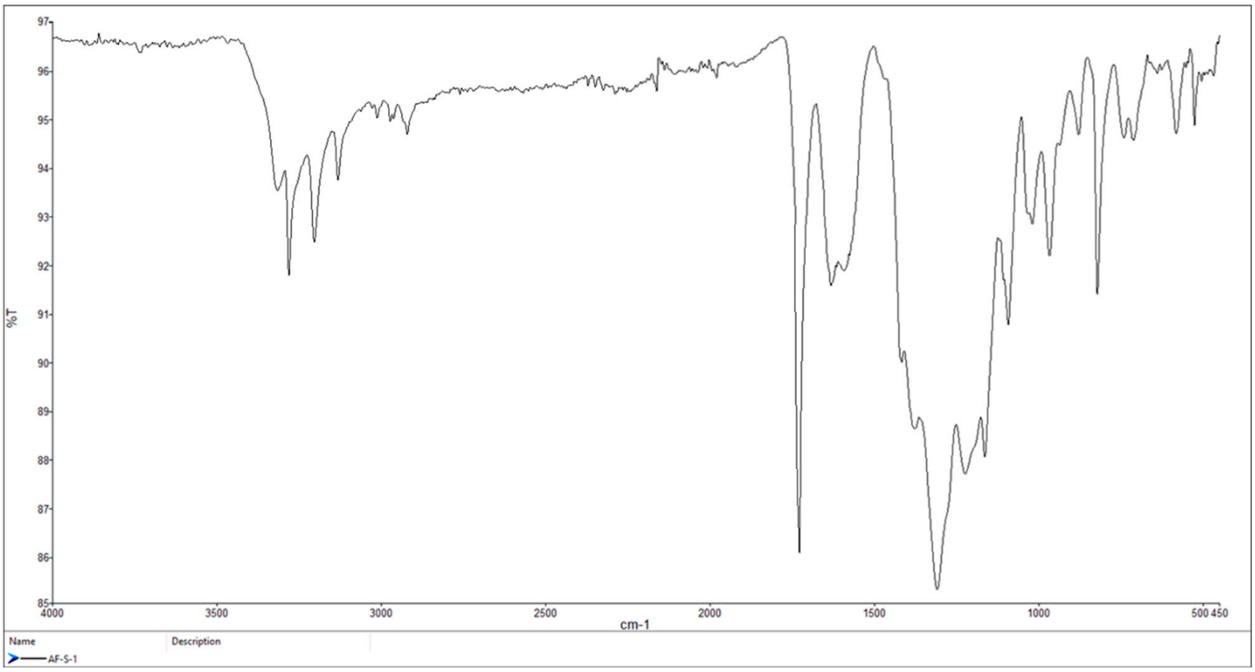

Fig. S3 IR spectrum of **Pd1-Pd3** complexes (KBr pellet, 298 K)
